# Supplementary material for: ε2-Phages Are Naturally Bred and Have a Vastly Improved Host Range in Staphylococcus aureus over Wild Type Phages
Source: Pharmaceuticals (Basel). 2021 Apr 2;14(4):325. doi: 10.3390/ph14040325 (PMC8065394; doi:10.3390/ph14040325)
Supplement: Supplementary file 1 [file pharmaceuticals-14-00325-s001.pdf]

pharmaceuticals

MDPI

Supplementary Materials

Table S1. Comparison of clonal complexes in *S. aureus* in this study with literature sources.

**Reference**	**This study**		**Kanjilal et al., 2018**	**Arias et al., 2017**	**Rasmussen et al., 2013**	**Rasmussen et al., 2013**	**Lüdicke et al., 2010**
**Country of Isolation**	**Germany**		**USA (Boston)**	**Latin America, Various Countries**	**Sweden**	**Sweden**	**Germany**
**Total Number of Strains**	**110**		**180**	**121**	**88**	**134**	**119**
**Source, Pathogenicity**	**Human Infections**		**Hospital Clinical Specimens**	**Bloodstream MRSA**	**Invasive *S.aureus* Only**	**All *S.aureus*, Non-Invasive and Infections**	**Infections of Bones, Prosthetic Implants**
**Conal Complex**	**# of Isolates in This Study**	**% of Total Strains**			**% of Strains**		
CC45	19	17%	4%		25%	26%	18%
CC30	16	15%	4%	9%	15%	23%	13%
CC8	15	14%	24%	27%	8%	7%	19%
CC22	9	8%		2%			3%
CC5	8	7%	31%	35%	12%	8%	7%
CC398	7	6%		1%			
CC25	6	5%			9%	8%	7%
CC101	3	3%					8%
CC12	3	3%					5%
CC15	3	3%	6%		14%	11%	6%
CC80	2	2%					1%
CC9	2	2%					2%
CC88	2	2%		2%			
CC6	2	2%					
CC239	2	2%		4%			
CC1	2	2%	8%	2%			
CC96	1	1%					1%
CC59	1	1%					1%
CC121	1	1%					3%
CC7	1	1%					3%
CC97	1	1%		1%			1%
CC772	1	1%					
CC60	1	1%					
CC395	1	1%					
CC49	1	1%					2%

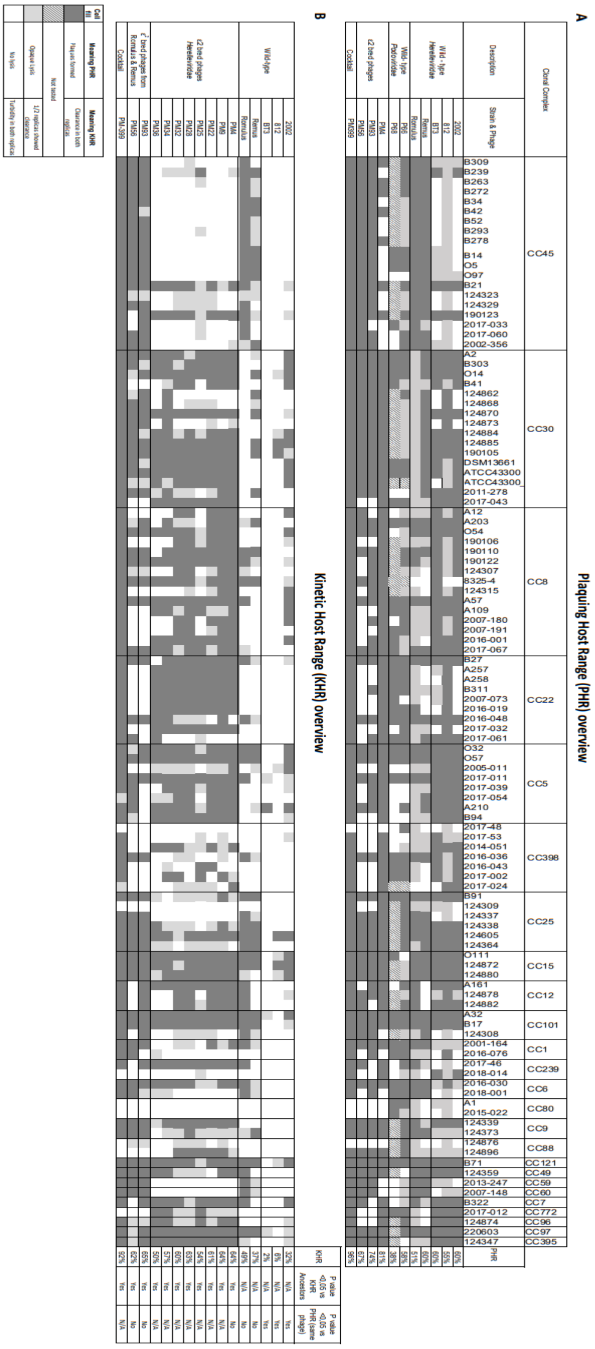

Figure S1. (**A**) Overview of the full plaquing host range of the ancestor phages. Each combination of phage and bacteria is represented by a square in different colors: White refers to no signs of lysis, light grey refers to opaque lysis and dark grey refers to plaque formation. (**B**) Kinetic host range overview of all phages described in the study. Each phage was tested twice against every bacterium in the panel of 110 *S. aureus* strains. Results are represented by a square in different colors: White indicates no clearance of bacteria after 24 h in any of the duplicates, light grey indicates clearance in one of the duplicates and dark grey indicates clearance in both duplicates tested. Striped line stands for not tested. In both parts of the figure, the phages tested are depicted on the left, grouped by originals or the experiment they were derived from. The bacterial strains are depicted in the top row, for both figures, grouped by clonal complex, with the MRSA/MSSA status shown in the second row of part B. Host range percentage is seen in the last columns of the table referring to each different phage. The last two columns refer to the statistical analysis, where KHR of bred phages was compared to the ancestors, and PHR and PHR values were compared between each phage, calculated by one-way ANOVA.

Table S2. Resistance rate of S. aureus *S. aureus* ATCC43300 (CC30/ST39-MRSA-II) in broth measured against different phages. LOD: limit of detection (1 cell in 10^{11}).

Replica Phage	Replica 1	Replica 2	Replica 3
PM4	<LOD	<LOD	N/A
PM93	<LOD	<LOD	N/A
PM-399	<LOD	<LOD	<LOD

Table S3. Mutational changes between PM56 and Romulus (JX846613.1).

Locus Tag	Module	Annotation	Mutation	Start	End	Romulus to PM56 DNA	Romulus to PM56 Amino acid
Reg	Unclear	regulatory	deletion	7512	7521	T to -	N/A
018	Structural	major capsid protein	single point	13,991	13,991	T to C	F25S
046	Structural	structural protein	single point	46,859	46,859	G to T	D300Y
046	Structural	structural protein	single point	47,513	47,513	G to A	E518K
081	Replication and transcription	intron-encoded endonuclease	insertion	78,909	78,911	TAT to TATAAT	N insertion after Y241
117	Unclear	pentapeptide repeat-containing protein	single point	97,750	97,750	G to A	M180I
161	Unclear	hypothetical protein	single point	114,767	114,767	A to C	N219K
187	Unclear	hypothetical protein	insertion	130,309	130,311	ATT to ATTATT	I insertion after I16

Table S4. Mutational changes between PM93 and Remus (JX846612.1).

Locus Tag	Module	Annotation	Mutation	Start	End	Remus to PM93 DNA	Remus to PM93 Amino acid
046	Structural	structural protein	single point	46859	46859	G to T	D383Y
046	Structural	structural protein	single point	47819	47819	C to A	Q703K
062	Replication and transcription	hypothetical protein	single point	61247	61247	G to T	V44L
117	Unclear	pentapeptide repeat-containing protein	single point	97692	97692	G to T	G161V
